# A Novel Time-Incremental End-to-End Shared Neural Network with Attention-Based Feature Fusion for Multiclass Motor Imagery Recognition

**DOI:** 10.1155/2021/6613105

**Published:** 2021-02-17

**Authors:** Shidong Lian, Jialin Xu, Guokun Zuo, Xia Wei, Huilin Zhou

**Affiliations:** ^1^College of Electrical Engineering, Xinjiang University, Urumqi 830047, China; ^2^Cixi Institute of Biomedical Engineering, Ningbo Institute of Materials Technology and Engineering, Chinese Academy of Sciences, Ningbo, Zhejiang 315201, China; ^3^Zhejiang Engineering Research Center for Biomedical Materials, Ningbo Institute of Materials Technology and Engineering, Chinese Academy of Sciences, Ningbo, Zhejiang 315300, China; ^4^University of Chinese Academy of Sciences, Beijing 100049, China

## Abstract

In the research of motor imagery brain-computer interface (MI-BCI), traditional electroencephalogram (EEG) signal recognition algorithms appear to be inefficient in extracting EEG signal features and improving classification accuracy. In this paper, we discuss a solution to this problem based on a novel step-by-step method of feature extraction and pattern classification for multiclass MI-EEG signals. First, the training data from all subjects is merged and enlarged through autoencoder to meet the need for massive amounts of data while reducing the bad effect on signal recognition because of randomness, instability, and individual variability of EEG data. Second, an end-to-end sharing structure with attention-based time-incremental shallow convolution neural network is proposed. Shallow convolution neural network (SCNN) and bidirectional long short-term memory (BiLSTM) network are used to extract frequency-spatial domain features and time-series features of EEG signals, respectively. Then, the attention model is introduced into the feature fusion layer to dynamically weight these extracted temporal-frequency-spatial domain features, which greatly contributes to the reduction of feature redundancy and the improvement of classification accuracy. At last, validation tests using BCI Competition IV 2a data sets show that classification accuracy and kappa coefficient have reached 82.7 ± 5.57% and 0.78 ± 0.074, which can strongly prove its advantages in improving classification accuracy and reducing individual difference among different subjects from the same network.

## 1. Introduction

The brain-computer interface (BCI) is a communication control system established between the brain and the external devices through the signals generated by brain activity. Creating direct communication between the brain and the external device, the system does not rely on muscles or peripheral nerves but the central nervous system [1]. Motor imagery (MI) is a psychological process in which an individual simulates the body movements. During the process of performing different MI tasks, when a certain area of the cerebral cortex is activated, the metabolism and blood flow of this area increase. Meanwhile, a simultaneous information processing will lead to an amplitude decrease or even block of EEG in its mu and beta spectrum oscillation. This electrophysiologic concept is called event-related desynchronization (ERD). In contrast, the phenomenon of a manifest amplitude increase of mu and beta oscillation, which appears in resting or inert states, is called event-related synchronization (ERS) [2].

The purpose of MI-BCI is to identify the imagined movements by classifying the electroencephalogram (EEG) characteristics of the brain, to control the external devices, such as robots [3, 4]. On the one hand, MI-BCI can help patients with severe dysfunction and establish communication channels with the outside world. On the other hand, to some extent, it can activate the brain region to promote the remodeling of the patient's central nervous system [5]. In contrast to the traditional rehabilitation training, it can improve the patient's subjective initiative to achieve the rehabilitation effect, which overcomes the defect of the passive and single means of traditional rehabilitation [6]. Therefore, MI-BCI has a growing potential value in the fields of motor function assist and motor neurorehabilitation. However, the high complexity and instability of the EEG signals make the feature extraction and pattern classification of signals very challenging.

The very important part of the MI-BCI system is how to classify the EEG characteristics of MI task correctly and convert it into external control instructions [4]. At present, the traditional MI-EEG signal feature extraction is mainly based on ERD/ERS in the *µ* band (8–12 Hz) and the *β* band (16–31 Hz), including signal bandpass filtering [7], autoregressive model [8], frequency domain statistics [9], phase-locking value (PLV) [10], wavelet transformation and wavelet-packet transformation [11, 12], information entropy [13], and common spatial pattern (CSP) [14]. Based on the above methods, Li et al. [15] used wavelet-packet transform (WPT) to analyze and rebuild the MI-EEG signals and extract the energy characteristics of the *µ* band and the *β* band. Zhang et al. [16] analyzed MI-EEG signals and extracted temporal and spatial features by using a one-versus-rest filter. However, traditional feature extraction relies on manual selection of specific frequency bands, and features are very limited, which may lose part of the EEG information. In addition, the pattern classification methods of MI-EEG signals include linear discrimination analysis (LDA) [17], bayesian linear discrimination analysis (BLDA) [18], logic regression (LR) [19], support vector machine (SVM) [20], and neural network (NN) [21]. The classification performance of these methods depends on the quality of feature extraction.

In more recent years, deep learning has made excellent achievements in the fields of speech recognition, image recognition, and natural language processing [22, 23]; it has been used as a good machine learning method in these fields for its advantages on self-learning of features [24–26]. Therefore, deep learning is also gradually used in the feature extraction and pattern classification of EEG signals, in some cases not only improve the accuracy, but also provide a new method to learn features from EEG data [27, 28]. For example, Tang et al. [29] investigated how convolution neural network (CNN) displayed spectral features of the series of MI-EEG samples. Yang et al. [30] used augmented CSP to extract the spatial features and CNN to learn deep structural features for MI-EEG classification on the BCI Competition IV data sets, which revealed that feature extraction no longer relied on manual ways. Tabar and Halici [31] proposed using a kind of deep learning method to classify MI-EEG signal patterns, CNN was used to extract features, and then stacked autoencoder (SAE) was used to classify the extracted features. However, MI-EEG signals are time series with strong time-varying characteristics, and CNN is not completely suitable for learning time-series features. Therefore, Lee and Choi [32] used continuous wavelet transform to extract the temporal-frequency features of MI-EEG signals and classified them by CNN. Zhou et al. [33] adopted a way based on wavelet-packet and long short-term memory (LSTM) neural network, which divided MI-EEG signals into several categories through the amplitude features and time-series information. An et al. [34] did some research on deep belief network (DBN) based on the restricted Boltzmann machine (RBM) linked up with fast Fourier transform (FFT) for MI-BCI pattern recognition, and the results were significantly better than those of traditional SVM-based algorithm. However, these methods simply extracted the temporal domain, frequency domain, or temporal-frequency domain features and did not fully extract the EEG signal features. Many other methods of deep learning have also been used in the recognition of MI-EEG signals, but the network structure is overly complex.

To sum up, all the above deep learning methods used in the recognition of MI-EEG signals do not take full advantages on self-learning of features, which still manually select features of specific frequency bands before pattern classification. Because the features selected manually are very limited and the objective function of feature extraction is different from that of pattern classification, it is easy to get information loss. What is more, multiclass MI-BCI classification mainly adopts splitting strategy. The whole process is extremely cumbersome and the classification accuracy is not high. Besides, the signal-to-noise ratio of MI-EEG signals is relatively low, and the data of the same person in the same task has randomness, instability, and individual variability, which makes the network trained with small-sample data sets have limitations. To reduce these limitations, a multimodal neural network is designed to form a novel end-to-end shared neural network in this paper. The main contributions are as follows:

1. For the sample size of BCI Competition IV 2a data sets is small, 1s time window is used to intercept the training data, and then autoencoder (AE) network is used to enlarge the sample size of all subjects' training data which is intercepted and merged in advance. It meets the requirement of a large amount of training data for neural network and effectively reduces the bad effect on signal recognition because of randomness, instability, and individual variability of EEG data.
2. To ensure the classification results of MI-EEG signals, a novel convolution neural network structure named the shallow convolution neural network (SCNN) is proposed to extract the different-dimension frequency-spatial domain features. Because of its simple structure and fewer parameters, the training model is not easy to overfitting. Furthermore, the EEG signals processed in the frequency-spatial domain are input into bidirectional long short-term memory (BiLSTM) network to extract the time-series features, so that the features in the MI-EEG signals are fully extracted. Finally, to reduce the redundancy of the fusion features and improve the classification accuracy, the attention model is introduced into the feature fusion layer to dynamically weight the extracted temporal-frequency-spatial domain features.
3. Through the proposed multimodal neural network, the training data of all subjects is used to train the end-to-end shared neural network, and it is tested by the test data of each subject and compared with the state-of-the-art methods in the MI-EEG recognition field to prove its higher classification accuracy and the minimum individual difference.

The structure of this paper is as follows: Section 1 is the introduction.  Section 2 describes the data sets and the details of the neural network method that we proposed. The experiment and its results are presented in Section 3. The discussion is presented in Section 4. Finally, Section 5 is the conclusion.

## 2. Materials and Methods

**Figure 1:**
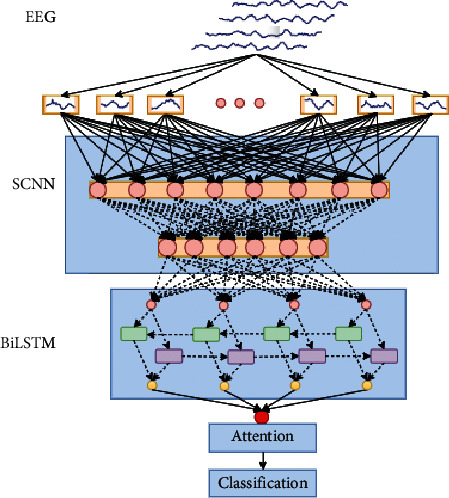
Our method of SCNN-BiLSTM network based on attention. The EEG signals are input to convolution layers and therefore preprocessed time series are generated. Moreover, the time series are input to BiLSTM cells for the exchange of information among different time points. And the attention mechanism module receives the output of BiLSTM cells, calculates weights for different time points, and outputs the ultimate result.

Different from images and videos, MI-EEG signals are time series with strong time-varying characteristics, which own a mass of data information while the amount of data is not large. In addition, its signal-to-noise ratio is low, and randomness, instability, and individual variability still exist in the process of signal acquisition. What is more, with the increase in the number of MI-EEG classifications, the strategy that multiclass task was split and the method that patterns were classified after feature extraction were both introduced in the past, but it is still difficult to improve the classification accuracy. To solve these problems, we put forward a method of an attention-based time-incremental end-to-end shared neural network, as shown in Figure 1. With a combination of SCNN network and BiLSTM network, and an attention model introduced into the feature fusion process, it is practicable for feature extraction and pattern classification in a step-by-step way for the temporal-frequency-spatial domain features of multiclass MI-EEG signals. This method is simply called the method of SCNN-BiLSTM network based on attention.

Before feature extraction and pattern classification are carried out in a step-by-step way, the training data of all subjects is expanded using AE network. Then, the different-dimensional frequency-spatial domain features which were abstract are extracted by different convolutional kernels of SCNN network, and the time-series features are extracted through BiLSTM network with time increments; after that, all the temporal-frequency-spatial domain features are combined with the attention mechanism. Finally, the above fusion features are input to output layer of the network for classifying. During the training process of this attention-based time-incremental end-to-end shared neural network, the convolution layers and the recurrent layers can receive the reverse propagation error of the output layer at the same time, and the gradient drop caused by the error will gradually spread to the front of the network. So, after many iterations, the network parameters are gradually updated, and the error will become smaller and smaller.

### 2.1. Data Description and Processing

**Figure 2:**
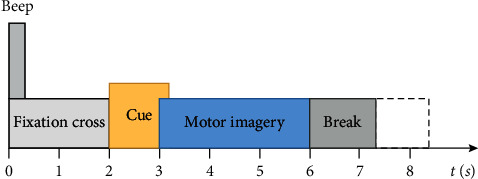
The paradigm timing scheme for MI-EEG data of BCI Competition IV 2a.

In this paper, the data sets are taken from four-class MI-EEG data of left hand, right hand, foot, and tongue in BCI Competition IV 2a in 2008 [35]. In the data sets, the EEG data of 9 subjects was recorded with 22 Ag/AgCl electrodes and labeled as A01–A09. The sample frequency is 250 Hz, band-pass filtering is set between 0.5 Hz and 100 Hz, and line noise is suppressed by a 50 Hz notch filter. Each experiment consists of two sessions. The first session is training and the second session is testing. One run contains 48 trials (12 for each of the four possible classes), resulting in 288 trials per session. The timing scheme of experimental data acquisition is shown in Figure 2.

Firstly, we select 2 s–6 s data from the training data sets *T* and intercept it with a time window of 1 s. After processing, the training data sets *T* of 9 subjects are merged and then enlarged with AE network. Secondly, to accelerate the convergence speed of the network, prevent interference caused by abnormal EEG data, and avoid unnecessary numerical problems, the segmented training data is standardized. What is more, to increase the stability of the network, the training data sets *T* with training labels are reordered randomly.

Particularly, in the process of data standardization, we standardize the EEG data based on the mean and standard deviation of the raw data, so as to avoid the influence of outliers and extreme values in the data through centralization. The processed EEG data conforms to the standard normal distribution with mean 0 and standard deviation 1.

The training sets and testing sets are both standardized before being input into the network as follows:

(1) $$\begin{array}{c} s_{\text{in}}\left(i\right)=\frac{s_{\text{in}}\left(i\right)-\text{mean}\left(s_{\text{in}}\right)}{\text{std}\left(s_{\text{in}}\right)}, \end{array}$$

(2) $$\begin{array}{c} p_{\text{in}}\left(i\right)=\frac{p_{\text{in}}\left(i\right)-\text{mean}\left(p_{\text{in}}\right)}{\text{std}\left(p_{\text{in}}\right)}, \end{array}$$

where *s*_in_ is the segmented training data and *p*_in_ is the segmented testing data.

And, during a single trial of BCI Competition IV 2a data, when *t* = 2 s, the prompt arrow appeared and lasted for 1.25 s; the subjects observed and imagined the corresponding action. When *t* = 3.25 s∼6 s, the subjects imagined the corresponding action. Because the EEG signals are instantaneous and susceptible to interference, in the process of motor imagery, the ERD/ERS characteristics of subjects' motor imagery EEG are uncertain during the transition from the preparation stage to the imagination stage, which is easy to cause invalid edge data. So, the method proposed in this paper is verified by selecting the motor imagery data of 4 s∼5 s with 0.75 s interval from *t* = 3.25 s in the final.

### 2.2. Data Expansion Based on Autoencoder

**Figure 3:**
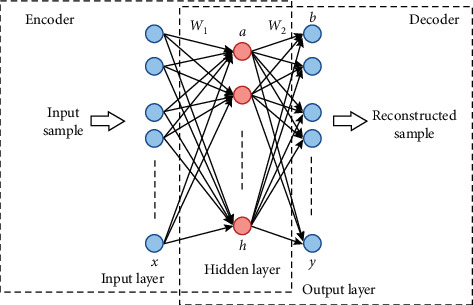
The structure of autoencoder neural network.

The deep learning methods need a large amount of data to train the network models. However, the data samples of BCI Competition IV 2a are small. In the meantime, the different periods and the size of the electrode caps during the data acquisition process make each subject's EEG signals have randomness and instability. Therefore, for the training sets, we select the data of 2 s∼6 s and use the 1 s time window to intercept and then use the autoencoder (AE) network to enlarge the data by generating the reconstructed data from the real training data in this paper, which is a three-layer neural network composed of an input layer, a hidden layer, and an output layer [36], as shown in Figure 3. After processing, it satisfies the need for a mass of training data of neural networks and improves the robustness of the network model, while effectively reducing the bad effect on signal recognition because of randomness, instability, and individual variability of EEG data.

In AE network, the output layer *y* has the same size as the input layer *x*, so *y* can be considered as an approximation of *x*. *f* and *g* represent the encoding and decoding functions, respectively. The encoding and decoding procedures are as follows:

(3) $$\begin{array}{c} h=f\left(x\right)=s_{f}\left(W_{1}x+a\right), \end{array}$$

(4) $$\begin{array}{c} y=g\left(h\right)=s_{g}\left(W_{2}h+b\right), \end{array}$$

where *h* denotes the hidden layer information, *W*_1_ denotes weight of the input layer to the hidden layer, and *W*_2_ denotes weight of the hidden layer to the output layer; *a* and *b* are biases of the hidden layer and the output layer, respectively; *s*_*f*_ and *s*_*g*_ are activation function of the encoding and decoding procedures, respectively. Here, both *s*_*f*_ and *s*_*g*_ adopt the sigmoid function. And, to simplify the calculation, let *W*_2_=*W*_1_^*T*^=*W*.

In this paper, the AE network firstly encodes the real training data to reduce the data dimension, and the important features of the data are retained through unsupervised learning. Then, the encoded data is decoded to obtain the reconstructed data. And, finally, the average value of the error reconstruction function between the reconstructed data and the real training data, namely, the loss cost function, is calculated to measure the similarity between them. The smaller the loss cost function is, the more similar the reconstructed data is to the real training data. However, in the process of network learning to obtain the parameter *θ*={*W*, *a*, *b*}, the value of the loss cost function will become smaller and smaller, which may result in overfitting. Therefore, we adopt cross-entropy in reconstruction error function to suppress overfitting to obtain an AE network with strong generalization ability. The reconstruction error function *R*(*x*, *y*) is defined as follows:

(5) $$\begin{array}{c} R\left(x,y\right)=-\sum_{i=1}^{n}\left[x_{i}\log\left(y_{i}\right)+\left(1-x_{i}\right)\log\left(1-y_{i}\right)\right]. \end{array}$$

For the entire training sets *S*={*x*_1_, *x*_2_,…, *x*_*m*_}, the overall loss cost function *J*(*θ*) is

(6) $$\begin{array}{c} J\left(\theta \right)=\frac{1}{m}\sum_{x\in s}R\left(x,y\right). \end{array}$$

The function *J*(*θ*) is minimized to obtain the parameter *θ* by the gradient descent method.

### 2.3. Shallow Convolution Neural Network

The structure of convolution neural network (CNN) is different from that of traditional hierarchical connections. The connections between neurons in CNN are not fully connected; what is more, the sharing weight of convolution kernel can reduce the complexity of the network model and reduce the weight parameters of network training, making it easier to train than the previous neural network [37].

**Figure 4:**
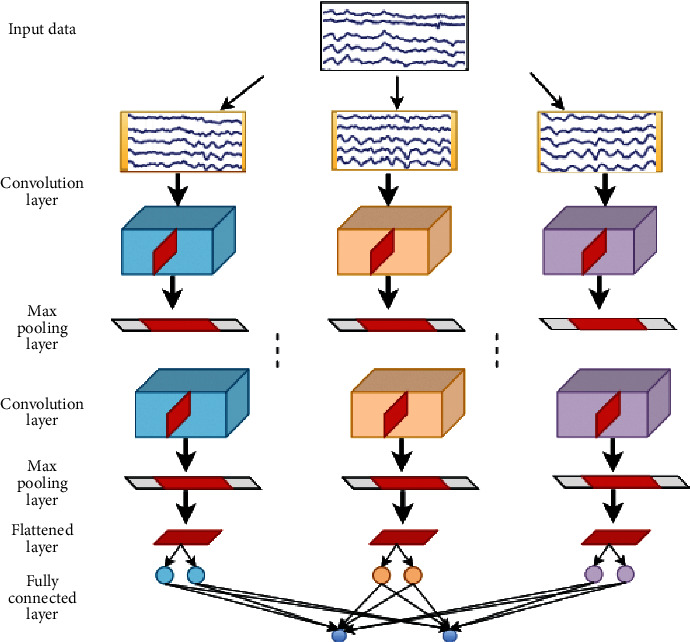
The structure of shallow convolution neural network. During the training, the input EEG data is *N∗f* (channel*∗*sample). Then, the maximum pooling layer is used to adjust the size of the convolution, which has kernels of 1*∗S* with a stride of 1*∗S*.

Nevertheless, compared with the information volume of images and videos, that of EEG signals is very small. Besides, it is a kind of nonstationary, random, very weak, and low signal-to-noise ratios signal with unstable waveform. When classifying EEG signals, we found that too many convolutional layers of CNN can easily lead to overfitting of the training model. Therefore, it is very crucial to structure a suitable CNN model. In this paper, the different-dimensional frequency-spatial domain features which were abstract are extracted by different convolutional kernels of shallow CNN network. The design of SCNN adopts the principle of the Visual Geometry Group (VGG) network [38]. It is a special CNN with a simple structure and few parameters. The training model is not easy to overfit and can directly extract the frequency-spatial domain features from the EEG data. The structure of SCNN is shown in Figure 4. The details of the structure used in this paper are shown in Table 1in Section 3.

The input is one-dimensional feature vector *X*=[*x*_1_, *x*_2_, *x*_3_,…, *x*_*N*_] with a length of *N* corresponding to EEG signals of *N* channels; the convolution layer is composed of *K* convolution kernels, the size of each convolution kernel is 1*∗S*, the coefficient of the convolution kernel is *w*_*k*_ ∈ *R*^*s*^,  *k*=1,2,…, *K*, and the output is *h*=[*h*_1_, *h*_2_, *h*_3_,…, *h*_*k*_] ∈ *R*^(*N* − *S*+1)*K*^, where

(7) $$\begin{array}{c} h_{k}=R\left(\text{conv}\left(w_{k}*x+b_{k}\right)\right), \end{array}$$

where *b*_*k*_ denotes the bias of the convolution kernel and *R* denotes the nonlinear activation function that adopts the Leaky ReLU function [39, 40].

(8) $$\begin{array}{c} f\left(x\right)=\left\{\begin{array}{cc} x, & x>0,\\ 0.01*x, & x\leq 0. \end{array}\right) \end{array}$$

In a single SCNN network structure, the network connects one or more full connection layers and a Softmax output layer after multiple convolution, pooling, and dropout layers. Supposing that the network has a total *L* layers, where the *L*_*M*_ layer is the full connection layer, the *L*_*L*_ layer is the final output layer, and the output number of cells is the number of classification categories *n*, the entire calculation process is as follows:

(9) $$\begin{array}{c} h^{l}=f\left(w^{l}X+b^{l}\right),\;L_{M}\leq l<L, \end{array}$$

(10) $$\begin{array}{c} a^{L}=w^{L}h^{l}+b^{L}, \end{array}$$

(11) $$\begin{array}{c} P\left(t||x\right)=\text{Soft }\max\left(a^{L}\right)=\frac{\exp\left(a_{t}^{L}\right)}{\sum_{t^{\prime }=1}^{n}\exp\left(a_{t^{\prime }}^{L}\right)},\;t=1,2,\ldots ,n, \end{array}$$

where *h*^*l*^ denotes the output of the convolution network's hidden layer information, *w*^*l*^ and *b*^*l*^ are the learning parameters of the network, *a*^*L*^ is the value which is not activated before the last output layer, and *P*(*t|x*) is a posterior probability judging whether the input *x* belongs to the category *t*. The label for each input's EEG signal category is *T*=[1,2,…, *n*]. For all samples in the training sets, cross-entropy Loss=(1/*n*)∑_*i*=1_^*n*^log  *p*(*t*=*i|X*) is taken as the objective function to optimize.

### 2.4. Bidirectional Long Short-Term Memory Network

EEG signals are not images in the traditional sense, but time series with a strong correlation in time. The SCNN network is not fully suitable for learning time-series features of EEG signals; however, recurrent neural network has certain advantages on that [41, 42]. Therefore, in this paper, BiLSTM network with time increments which is a kind of recurrent neural network is connected in series before the full connection layer and after multiple convolution, pooling, and dropout layers of the SCNN network. Different from the traditional unidirectional LSTM network, the BiLSTM network improves on network structure so as to solve the gradient disappearance well and more fully extracts the information of each time point, which is suitable for EEG processing in temporal domain. The input at each moment of BiLSTM network comes from the information transmitted by the hidden layers in the forward and backward directions, and then the network combines the output of the forward and backward hidden layers to obtain its final output of each moment.

**Figure 5:**
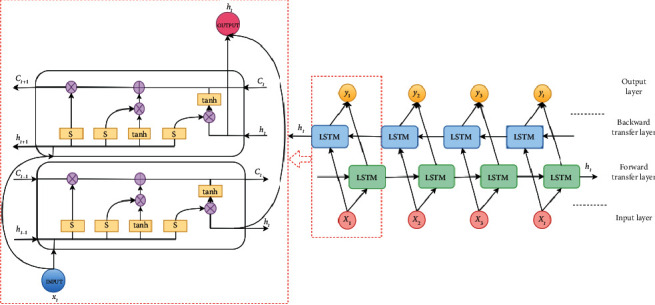
The structure and principle of bidirectional long short-term memory network with time increments. “*S*” represents sigmoid operator; “tanh” signifies hyperbolic tangent operator. “*C*_*t*_” represents the state of BiLSTM cell at *t* moment.

In this paper, to reduce the local convergence caused by fewer layers and the gradient disappearance caused by too many layers, the two-layer BiLSTM network is designed to converge more quickly and effectively reduce the gradient disappearance caused by too deep propagation between layers. The network's structure and principle are shown in Figure 5.

BiLSTM network is a unidirectional LSTM network when it is performing forward calculation, and the forward calculation requires the input data before the current time. The forward calculation of the network is as follows:

(12) $$\begin{array}{c} \overset{⟶}{h_{t}}=W_{x_{h}^{\to }}x_{t}+W_{\overset{\to }{h}\overset{\to }{h}}h_{t-1}^{\to }+b_{h}^{\to }, \end{array}$$

When it is performing backward calculation, it is associated with the future input data after the current time. The backward calculation of the network is as follows:

(13) $$\begin{array}{c} {\overset{\leftarrow }{h}}_{t}=W_{x_{h}^{\leftarrow }}x_{t}+W_{\overset{\leftarrow }{h}\overset{\leftarrow }{h}}h_{t-1}^{\leftarrow }+b_{h}^{\leftarrow }. \end{array}$$

The LSTM networks in the forward and backward directions maintain the state information of their own network, respectively, there is no connection between them, and the unfolded diagram of the network is not a circular structure. Having superimposed the state information coming from both directions simultaneously, then the output layer can be calculated. The overall calculation of the network is as follows:

(14) $$\begin{array}{c} y_{t}=W_{\overset{⟶}{h}y}\overset{⟶}{h_{t}}+W_{\overset{\leftarrow }{h}y}{\overset{\leftarrow }{h}}_{t}+b_{y}, \end{array}$$

where *x*_*t*_ and *y*_*t*_ denote the input and the output layers, respectively; *W* and *b* represent the network's weights and biases, respectively.

When BiLSTM network is combined in series with SCNN network, the calculation method for the whole network is as follows:

(15) $$\begin{array}{c} h_{\text{scnn}}^{l}=f\left(w^{l}X+b^{l}\right),\;L_{M}\leq l<L, \end{array}$$

(16) $$\begin{array}{c} a_{\text{SCNN}}^{L}=w^{L}h_{\text{scnn}}^{l}+b^{L}, \end{array}$$

(17) $$\begin{array}{c} a_{\text{BiLSTM}}^{t}=W_{\overset{⟶}{h}a}\overset{⟶}{h_{t}}+W_{\overset{\leftarrow }{h}a}{\overset{\leftarrow }{h}}_{t}+b_{a}n, \end{array}$$

(18) $$\begin{array}{c} a_{\text{BiLSTM}}=U_{t}a_{\text{BiLSTM}}^{t}, \end{array}$$

(19) $$\begin{array}{c} h_{\text{FFL}}=W_{\text{FFL}}\left(a_{\text{SCNN}}^{L}+a_{\text{BiLSTM}}\right)+b_{\text{FFL}}, \end{array}$$

(20) $$\begin{array}{c} P\left(t||x\right)=\text{Soft }\max\left(h_{FFL}\right)=\frac{\exp\left(h_{FFLt}\right)}{\sum_{t^{\prime }=1}^{n}\exp\left(h_{FFLt^{\prime }}\right)},\;t=1,2,\ldots ,n, \end{array}$$

where *h*_scnn_^*l*^ is the hidden layer information that is output from the whole SCNN network. After a linear transformation, it is found that *a*_SCNN_^*L*^ is a set of effective EEG features for different categories extracted by the network from the input EEG data. Having the hidden feature of EEG signals in temporal dimension, *a*_BiLSTM_ is the synthesis of *a*_BiLSTM_^*t*^ output from BiLSTM network of all time nodes, and it can largely reflect the abnormal changes of EEG signals in the temporal dimension. Then, the frequency-spatial domain features extracted by SCNN network and the time-series features extracted by BiLSTM network are synthesized in the feature fusion layer and the fusion features of the temporal-frequency-spatial domain are obtained. Finally, the highly abstract features that have undergone multiple convolutions and cycles will be fused after a linear transformation. The relative proportions of the “good” and “bad” features are adjusted by learning weights from the training data; then, the proportions are sent into the output layer for the probability calculation of each category.

The above feature fusion is only synthesized in the direction of one-dimensional vector. The frequency-spatial domain features extracted by SCNN network and the time-series features extracted by BiLSTM network will have some redundancy. The fusion features, mechanically synthesized, will be redundant which will slow down the network training speed and then spoil final classification effect. Therefore, in this paper, attention mechanism is added to process the fusion features.

### 2.5. Attention Mechanism

In cognitive science, to reasonably use the finite resources of visual information processing, humans usually choose to ignore part of the information and pay attention to the more critical part of all the information; that is to say, the brain's attention is focused on the specific visual area; this mechanism is called attention mechanism [43]. In this paper, the feature fusion process is optimized through the attention mechanism. The frequency-spatial domain features extracted by SCNN network and the time-series features extracted by BiLSTM network are fused and the important degree of the fusion feature is calculated to obtain the effective attention, so as to realize the automatic classification of MI-EEG signals by more effectively fusing temporal-frequency-spatial domain features.

**Figure 6:**
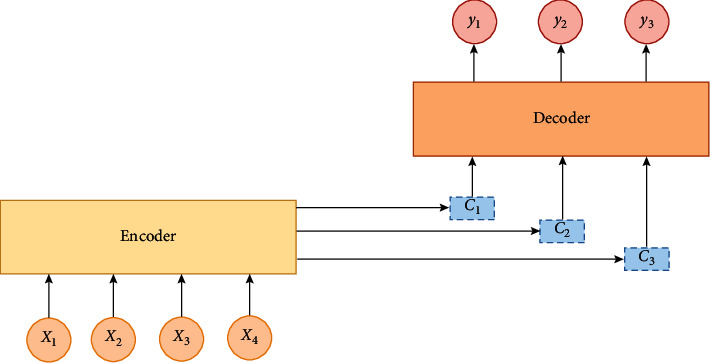
Encoder-decoder process.

In traditional sequence-to-sequence learning, the encoder-decoder structure is often used for learning, as shown in Figure 6.

The encoder encodes the input sequence to get the intermediate state information *C* and then uses the intermediate vector as the input of the decoder to get the output of each sequence at the decoding end. The overall process is as follows:

(21) $$\begin{array}{c} P\left(y_{t}|\left\{y_{1},y_{2},\ldots ,y_{t-1}\right\},c\right)=g\left(y_{t-1},s_{t},c\right). \end{array}$$

**Figure 7:**
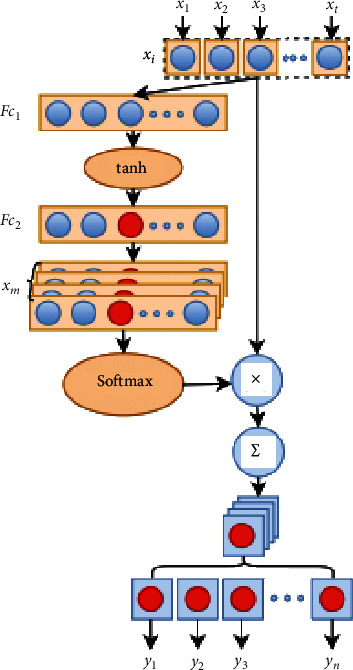
The structure of attention model. In the attention model, there are two fully connected layers *F*_*c*1_ and *F*_*c*2_ and a hyperbolic tangent function tanh. The first fully connected layer calculates *x*_*i*_′=∑_*k*=1_^*t*^*w*_*ik*_*x*_*k*_+*b*_*i*_ from each input feature vector *x*_*i*_(*i*=1,…, *t*), where *b*_*i*_ is the bias. The hyperbolic tangent function performs a nonlinear transformation on *x*_*i*_′ to obtain *x*_*i*_^″^=tanh(*x*_*i*_′). And, after the second full connected layer, *x*_*mi*_=∑_*k*=1_^*t*^*w*_*mik*_*x*_*k*_^″^+*b*_*mi*_,  (*m*=1,…, *n*;  *i*=1,…, *t*) is obtained. Then, Softmax calculates the weight coefficient *α*_*mi*_ of each feature in*x*_*m*_sequences. Therefore, the attention model outputs important features for each input feature vector *x*_*i*_, which is *y*_*m*_=∑_*i*=1_^*t*^*α*_*mi*_*x*_*i*_,  (*m*=1,…, *n*).

The output at each moment uses the same context semantic vector *C*, but, in the process of sequence encoding and decoding, we hope that the context semantic vector for each moment's output is an appropriate vector, so the attention mechanism is introduced to select the appropriate context semantic vector according to the output of different moments. The attention model is shown in Figure 7.

The decoding process for the attention model is as follows:

(22) $$\begin{array}{c} P\left(y_{i}|\left\{y_{1},y_{2},\ldots ,y_{i-1}\right\},x\right)=g\left(y_{i-1},s_{i},c_{i}\right), \end{array}$$

(23) $$\begin{array}{c} s_{i}=f\left(s_{i-1},y_{i-1},c_{i}\right), \end{array}$$

where *c*_*i*_ is the added attention; its role is to associate the output with the relevant input and to calculate the correlation *a*_*ij*_ between the current output and all inputs; then,

(24) $$\begin{array}{c} c_{i}=\sum_{j=1}^{T_{x}}a_{ij}h_{j}, \end{array}$$

where *h*_*j*_ is the hidden layer information at the position *j* of the encoder's input. In this paper, the SCNN outputs EEG signal with frequency-spatial domain features as the input of the encoder BiLSTM; that is, $h_{j}=\left[\overset{⟶}{h_{j}},\overset{⟵}{h_{j}}\right]$, where the forward and backward of the hidden layer information are synthesized. Weight *a*_*ij*_ identifies the relevancy of the input sequence to the current output sequence, which is a normalized probability value, meaning the probability of the relationship between item *j* of the input and the output at the current moment. And weight *a*_*ij*_ is defined as

(25) $$\begin{array}{c} a_{ij}=\frac{\exp\left(e_{ij}\right)}{\sum_{k=1}^{T_{x}}\exp\left(e_{ik}\right)}, \end{array}$$

(26) $$\begin{array}{c} e_{ij}=a\left(s_{i-1},h_{j}\right). \end{array}$$

The definition of *a*_*ij*_ introduces the symbol *e*_*ij*_ as a feedforward neural network, which is jointly determined by the state information *s*_*i*−1_ of the hidden layer at the decoding end and *h*_*j*_ of the hidden layer at the encoding end. In the SCNN-BiLSTM network based on attention that we designed above, the attention module is an additional neural network, which can give different weights to each part of the fusion features and is more sensitive to the classification target; it can effectively enhance the performance of the whole neural network in a natural way.

## 3. Experiment and Results

We refine the model of SCNN-BiLSTM network based on attention that is designed in Section 2 and then train and test the model to verify its superiority in MI-EEG multiclass recognition. The model is trained and tested on the Intel 3.6 GHz Core i7-10700F CPU and 16 GB RAM NVIDIA GeFore RTX 2060 GPU.

The details of the network model are shown in Table 1.

For the above deep neural network model, the minibatch gradient descent method is used for network training. To accelerate the attenuation of the network, the Adam optimizer is used for the network model, so that the model converges to the optimal value [44]. In the training of the model, the setting of the learning rate and the selection of the minibatch size affect the model's final accuracy and training speed, so in this paper we fix the other parameters of the model, constantly change the size and attention frequency of the learning rate, set the minibatch size in different sizes, and compare the final accuracy of the model. After multiple comparative experiments, the minibatch size takes 200 and the initial value of the learning rate is set to 0.001.

While training the neural network, random dropout and padding strategies are used. Among them, the random dropout strategy for the SCNN network can prevent the network model from overfitting the training data, while the padding strategy makes the output size of the convolution layer equal to the input size to prevent the loss of feature size [45]. In this paper, the random dropout parameter *P* is 0.2.

**Figure 8:**
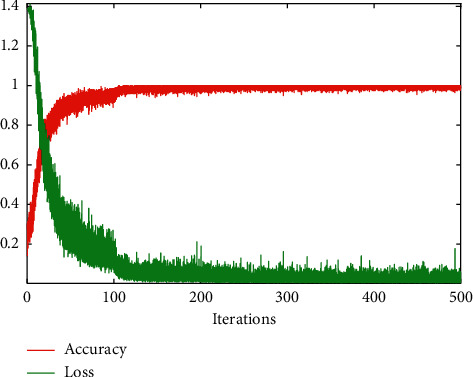
The relationship between the training loss rate, accuracy curve, and iterations. We repeated the training process of the network model for 500 times.

Figure 8 shows the training loss rate and accuracy curve of the neural network model after 500-time repeated training. It can be seen that, after 240 iterations, the training accuracy curve converges to 0.9 and the training loss rate is about 0.1.

**Figure 9:**
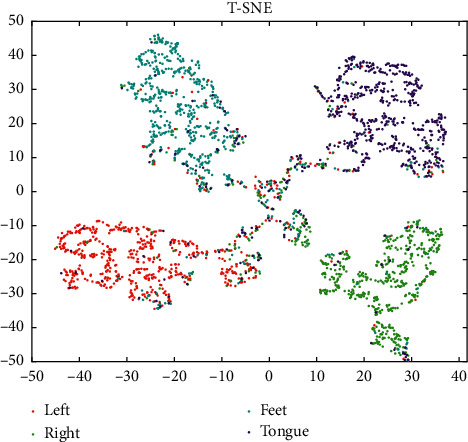
Visualizations of the high-dimensional data of four-class MI-EEG features.

We test the trained network model by the test data sets *E*; each point of the high-dimensional data of four-class MI-EEG features is assigned on the low-dimensional map and is avoided to concentrate in the center of the map, so as to form a scatter plot of T-distributed Stochastic Neighbor Embedding (T-SNE) [46], as shown in Figure 9. In the T-SNE scatter plot, the classification categories are represented by different colors, and it can be seen that all categories are clearly separated, but there is also some data hard to identify, which may be caused by interference during data acquisition.

**Figure 10:**
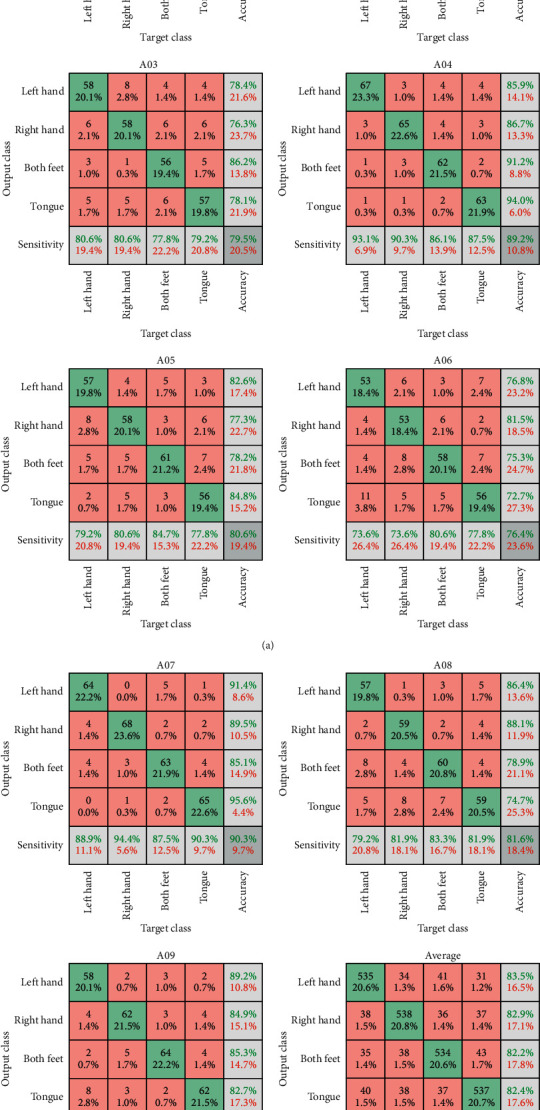
Confusion matrix of the trained network model. The classification accuracy rate of each subject in BCI Competition IV 2a (A01–A09). The average classification accuracy rate of all subjects (average). Take the confusion matrix of A01 as an example. The first column indicates that, in a total of 72 testing samples from 72 left-hand trials, the number of testing samples is 64 whose model predicted value of classification category is left hand, and the number of testing samples is 8 whose model predicted value of classification category covers right hand, feet, and tongue; that is, TP = 64 and FN = 8; then, TPR = 64/72 = 88.9%. The second to fourth columns are the same. The first row indicates that, in a total of 72 testing samples from 64 left hand, 2 right hand, 5 feet, and 1 tongue trials, all the testing samples' model predicted values of classification category are left hand; that is, TP = 64 and FP = 8; then, PPV = 64/72 = 88.9%. The second to fourth rows are the same. The main diagonal indicates that the total number of model's correct judgments is 64 + 62+64 + 64 = 254 times, while the total model prediction results of testing samples are 288, so, ACC = 254/288 = 88.2%.

Further, the network model which has been trained is measured by indicators such as accuracy, precision, sensitivity and specificity, and these indicators are calculated as follows:

(27) $$\begin{array}{c} \text{ACC}=\frac{{\text{TP}}_{n}+{\text{TN}}_{n}}{{\text{TP}}_{n}+{\text{TN}}_{n}+{\text{FP}}_{n}+{\text{FN}}_{n}}, \end{array}$$

(28) $$\begin{array}{c} \text{PPV}=\frac{{\text{TP}}_{n}}{{\text{TP}}_{n}+{\text{FP}}_{n}}, \end{array}$$

(29) $$\begin{array}{c} \text{TPR}=\frac{{\text{TP}}_{n}}{{\text{TP}}_{n}+{\text{FN}}_{n}}, \end{array}$$

(30) $$\begin{array}{c} \text{TNR}=\frac{{\text{TN}}_{n}}{{\text{TN}}_{n}+{\text{FP}}_{n}}, \end{array}$$

where TP represents the number of testing samples whose real value and model predicted value of classification category are both true, TN represents the number of testing samples whose real value and model predicted value of classification category are both negative, FP represents the number of testing samples whose real value of classification category is negative but their model predicted value is positive, and FN represents the number of testing samples whose real value of classification category is positive but their model predicted value is negative. Accuracy (ACC) is the proportion of the total number of model's correct judgments in the total model prediction results of testing samples. Precision (PPV) is the proportion of the number of model's correct judgments in the model prediction results of testing samples whose predicted value of classification category is positive. Sensitivity (TPR) is the proportion of the number of model's correct judgments in the model prediction results of testing samples whose true value of classification category is positive. Specificity (TNR) is the proportion of the number of model's correct judgments in the model prediction results of testing samples whose true value of the classification category is negative. *n* denotes the classification categories. The trained model classifies the test data of subjects in BCI Competition IV 2a; the classification accuracy rate of each subject and average classification accuracy rate of all subjects are shown in Figure 10.

## 4. Discussion

Our method of SCNN-BiLSTM network based on attention in this paper is compared with the methods in the literature [16, 31, 47–52] and the classification accuracy of each method is measured by kappa coefficient. In the classification problem, the higher the kappa coefficient [53], the higher the classification accuracy. The kappa coefficient is calculated as follows:

(31) $$\begin{array}{c} \text{Kappa}=\frac{\text{ACC}-\left(1/C\right)}{1-\left(1/C\right)}, \end{array}$$

where *C* denotes the number of known categories and ACC is the average classification accuracy.

For analyzing the literature methods, see Table 2. The literature in [47] proposed the Filter Bank Common Spatial Pattern (FBCSP) method to extract features of MI-EEG signals and adopted the “one-versus-rest” multiclassification mechanism, which won the 2008 International Brain-Computer Interface Competition. The literature in [48] proposed an automatic method for the classification of general artifactual source components, which was a kind of Independent Component Analysis (ICA) for artifact removal in MI-EEG signals, and the classification accuracy was 69.7 ± 14.2%. The literature in [49] proposed a method which spectral regression kernel discriminant analysis (SRKDA), with a classification accuracy of 78.4 ± 14.0%. The literature in [50] proposed a method which combined CSP and Local features-scale Decomposition (LCD) to extract features of MI-EEG signals, with classification accuracy of 80.2 ± 8.10%. The literature in [51] proposed a method of adaptive Stacked Regularized Linear Discriminant Analysis (SRLDA) to analyze the temporal, spatial, and spectral information of MI-EEG signals. The results showed that the adaptive SRLDA method was superior to the method of Data Space Adaptation (DSA) based on Kullback-Leibler divergence. However, the above-mentioned literature methods completely rely on human's current cognition of EEG signals and require relevant professional knowledge in the process of feature extraction, which makes the feature extraction too complicated and the classification effect poor. The literature in [52] proposed a method based on the combination of wavelet transformation and 2-layer CNN network, with classification accuracy of 81.2 ± 28.5%. The literature in [16] proposed using “one-versus-rest” Filter Bank Common Spatial (OVR-FBCSP) mode to extract features of MI-EEG signals primarily; then, CNN and LSTM networks were applied to reextract and classify those primary processing features. The classification accuracy was 83.0 ± 8.34%. Although these methods have achieved some accomplishments, they did not fully utilize the advantage of deep learning's self-learning characteristics and still followed the idea of manually extracting features first and then classifying patterns. Tabar and Halici [31] and Amin et al. [37] proposed new deep learning methods of feature extraction and pattern classification for MI-EEG signals, but the classification accuracy was not high, which was 66.2 ± 11.2% and 74.5 ± 10.1%, respectively.

**Figure 11:**
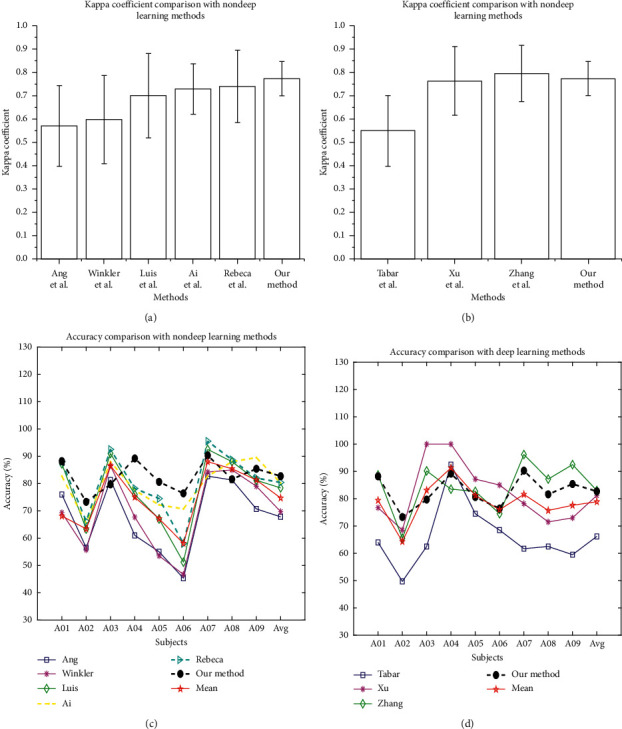
Comparison results of the accuracy and kappa coefficient. (a, c) The comparison results between the traditional nondeep learning methods and our method in this paper. (b, d) The comparison results between the deep learning methods and our method in this paper. It is obvious from (a, b) that our method has the minimum individual difference among different subjects, and also as can be seen from (c, d) our method has greatly improved the overall accuracy of all 9 subjects.

In this paper, we propose a method of an attention-based time-incremental end-to-end shared neural network. After extracting the frequency-spatial domain features by SCNN network and extracting the time-series features by BiLSTM network with time increments, the method effectively learns the temporal-frequency-spatial domain features of MI-EEG signals. Finally, an attention mechanism is added to the network feature fusion layer, and the extracted temporal-frequency-spatial domain features are dynamically weighted to reduce the redundancy of the fusion features and improve the classification accuracy rate to 82.7 ± 5.57%. The results of comparison between our method and the literature methods are shown in Figure 11. It is obvious from (a) and (b) that, compared with the nondeep learning methods and deep learning methods, our method has the minimum individual difference among different subjects, and also as can be seen from (c) and (d) the classification accuracy of each subject is greater than 73.3%. In other words, our method has greatly improved the overall accuracy of all 9 subjects. Therefore, our method is more suitable for the multiclass recognition of MI-EEG signals that are short-time series and is very effective in improving the overall classification and recognition.

## 5. Conclusions

In this paper, we propose an attention-based time-incremental end-to-end shared neural network which is essentially an end-to-end trainable model formed through the unification of SCNN network, BiLSTM network, and attention mechanism. With this end-to-end shared neural network, the feature extraction and pattern classification of MI-EEG signals are performed in a step-by-step way, which effectively improves the accuracy and robustness of EEG recognition.

In much research of deep neural network methods for EEG signals, the amount of training data is not enough for the network's training, and EEG signals are treated as images in usual, which may lead to the loss of information about time. Moreover, a simple network stacking can cause redundancy of features. So, to solve these issues, the method in this paper is divided into the following steps: first, a combination of all the sample training data followed by an increase in sample number using autoencoder meets the needs of a mass of training data for deep learning while effectively reducing the bad effect on signal recognition because of randomness, instability, and individual variability of EEG data. Second, BiLSTM network with time increments is connected in series with SCNN network, so that feature extraction of MI-EEG in its frequency-spatial domain and temporal domain successively uses SCNN network and BiLSTM network, which can make the features of MI-EEG in its temporal-frequency-spatial domain fully learned and ensure the classification results of MI-EEG signal. Third, the attention mechanism introduced into the dynamic weighted feature fusion of MI-EEG reduces the redundancy of the fusion features and improves the classification accuracy.

The results of comparison with the traditional nondeep methods and deep learning methods have shown the effectiveness of this end-to-end shared neural network that we proposed. The method is more suitable for the multiclass recognition of MI-EEG signals that are short-time series. It has the minimum individual difference among different subjects and is very effective in improving the overall classification and recognition of subjects.

In the near future, we will continue keeping focus on the research of information in raw MI-EEG data. Through the analysis of the irregularities of both distribution structure of EEG channels and EEG data, we will try to learn more about the algorithms of feature extraction, feature fusion, and pattern classification to recognize motor imagery tasks more compared to four classes and improve the classification accuracy while reducing the individual difference of the same network model in different subjects. What is more, we will also try to deploy the combination of brain-computer-interface and limb rehabilitation robot in an online system.

## Figures and Tables

**Table 1:** The details of the network model.

Layers	Type

Conv 1	Convolution 1 × 5
Norm 1	Batch normalization
Pool 1	Max pooling 1 × 2
Conv 2	Convolution 1 × 3
Norm 2	Batch normalization
Pool 2	Max pooling 1 × 2
Drop 2	Dropout layer
Conv 3	Convolution 1 × 3
Norm 3	Batch normalization
Drop 3	Dropout layer
Conv 4	Convolution 1 × 3
Norm 4	Batch normalization
Flattened	Flattened layer
LSTM 1	BiLSTM layer 32
LSTM 2	BiLSTM layer 20
Fc 5	Fully connected layer
Drop 5	Dropout layer
Attention	Attention layer
Fc 6	Fully connected layer
Classification	Softmax layer

**Table 2:** Comparison of the accuracy and kappa coefficient between the method in this paper and the methods in the literature.

Methods	Statistics	Subjects									Mean ± sd
		A01	A02	A03	A04	A05	A06	A07	A08	A09	

Tabar and Halici [31]	Accuracy kappa	64.0% 0.520	49.7% 0.330	49.7% 0.330	92.5% 0.910	74.5% 0.660	68.5% 0.580	61.7% 0.490	62.5% 0.500	59.5% 0.460	66.2 ± 11.2% 0.550 ± 0.152
Ang et al. [47]		76.0% 0.680	56.5% 0.420	81.3% 0.750	61.0% 0.480	55.0% 0.400	45.3% 0.270	82.7% 0.770	81.3% 0.750	70.7% 0.610	67.8 ± 12.9% 0.570 ± 0.173
Winkler et al. [48]		69.3% 0.590	55.7% 0.410	86.5% 0.820	67.7% 0.570	53.5% 0.380	46.7% 0.290	84.2% 0.790	85.0% 0.800	79.0% 0.700	69.7 ± 14.2% 0.600 ± 0.189
Nicolas-Alonso et al. [49]		87.2% 0.830	63.3% 0.510	91.0% 0.880	76.0% 0.680	67.0% 0.560	51.2% 0.350	92.5% 0.900	88.0% 0.840	89.5% 0.750	78.4 ± 14.0% 0.700 ± 0.180
Ai et al. [50]		82.7% 0.770	65.5% 0.540	88.0% 0.840	77.5% 0.700	72.2% 0.630	70.7% 0.610	82.7% 0.770	88.0% 0.840	89.5% 0.860	80.2 ± 8.10% 0.730 ± 0.108
Nicolas-Alonso et al. [51]		88.0% 0.840	66.2% 0.550	92.5% 0.900	78.2% 0.710	74.5% 0.660	58.0% 0.440	**95.5%** **0.940**	**88.7%** **0.850**	82.0% 0.760	80.4 ± 11.8% 0.740 ± 0.157
Xu et al. [52]		76.7% 0.690	68.5% 0.580	**100%** **1.000**	**100%** **1.000**	**87.2%** **0.830**	**85.0%** **0.800**	78.2% 0.710	71.5% 0.620	73.0% 0.640	81.2 ± 28.5% 0.750 ± 0.147
Zhang et al. [16]		87.7% 0.850	65.5% 0.540	88.2% 0.870	83.5% 0.780	82.7% 0.770	74.5% 0.660	93.2% 0.920	87.2% 0.830	**91.5%** **0.900**	83.0 ± 8.34% 0.800 ± 0.120
SCNN-BiLSTM network based on attention		**88.2%** **0.840**	**73.3%** **0.650**	79.7% 0.730	89.2% 0.870	80.6% 0.740	76.4% 0.690	90.3% 0.870	81.6% 0.760	85.4% 0.810	**82.7** **±** **5.57%** **0.780** **±** **0.074**

## Data Availability

The BCI Competition IV data set 2a is available at http://www.bbci.de/competition/iv/.
